# Multimodal Pain Management in Orthopedic Surgery

**DOI:** 10.3390/jcm11216386

**Published:** 2022-10-28

**Authors:** Aparna Chunduri, Amit Kumar Aggarwal

**Affiliations:** 1 Private Practice, Odessa, FL 33556, USA; 2 Department of Anesthesiology, The University of Texas Medical Branch Galveston, Galveston, TX 77555, USA

**Keywords:** multimodal analgesia, orthopedic surgery, pain management, anesthesiology, opioids management

## Abstract

Background: Orthopedic surgery typically results in moderate to severe pain in a majority of patients. Opioids were traditionally the primary medication to target mechanisms of pain transmission. Multimodal analgesia has become a preferred method of pain management in orthopedic practice. Utilizing more than one mode to address post-surgical pain by recruiting multiple receptors through different medications accelerates the recovery process and decreases the need for opioids. By implementing effective analgesic techniques and interventions, this practice, in turn, decreases the usage of perioperative opioids, and in the long term, prevents addiction to pain medications and risk of opioid overdose. In orthopedic surgeries, previous studies have found that multimodal analgesia has reduced early opioid usage in the postoperative course. Pain is the result of direct injury to the nervous system, with a wide variety of chemicals directly stimulating or sensitizing the peripheral nociceptors. The pathophysiology behind the mechanism of post-surgical pain, along with the importance of preoperative, perioperative, and postoperative pain regimens are emphasized. A brief overview of pain medications and their properties is provided. These medications are further categorized, with information on special considerations and typical dosage requirements. Pain management should address both neuropathic and subjective types of pain. Effective pain control requires constant reassessment with individualized strategies. Conclusion: By focusing on multimodal analgesia, anesthesiologists can now utilize newer techniques for postoperative pain relief from orthopedic surgery, with better short-term and long-term outcomes for the patient.

## 1. Introduction

Deaths due to drug overdose have become an epidemic in the United States [1]. Between 1999 and 2016, more than 600,000 people died from drug overdoses, mostly related to opioids prescribed for pain [2]. Nearly half of the patients who take opioids for at least 3 months remain on opioids 5 years later and are likely to become lifelong users [3,4,5,6]. Orthopedic procedures are considered one of the most painful procedures a patient can undergo.

According to the revised International Association for the Study of Pain (IASP) definition, pain is “An unpleasant sensory and emotional experience associated with, or resembling that associated with, actual or potential tissue damage” [7]. Orthopedic surgery, especially total joint replacement, results in moderate to severe pain in a majority of patients. Improvements in pain management have been amongst the most substantial advancements in the practice of total joint replacement surgery [8]. Appropriate treatment of pain in these patients promotes healing, shortens length of recovery, and improves quality of life after surgery. Pain has become the “fifth vital sign” in the view of the Joint Commission on Accreditation of Healthcare Organizations (JCAHO) and demands consideration in the care of all patients. Pain not only needs to be considered during the discharge decision but also during the entire inpatient and outpatient course [9]. Pain demands treatment, and failure to provide adequate treatment can result in medico legal action [10]. Ever since Professor Henrik Kehlet introduced the concept of Enhanced Recovery After Surgery (ERAS), multimodal analgesia became a preferred method of pain management. It includes preoperative, perioperative, and post-operative components and calls for a multidisciplinary collaboration between patients, surgeons, anesthesiologists, physiotherapists, occupational therapists, and nursing staff. Using more than one mode, including psychotherapy, physical therapy, regional anesthesia, local injections, and nonopioid medications, to address post-surgical pain results in superior pain control and accelerates the recovery process with less need for opioids, therefore decreasing the potential risk of abuse. Previous literature has shown that multimodal analgesia has decreased the length of stay and pain in the first 24 h after foot and ankle surgery. By adding periarticular injections to standard pain control for hip hemiarthroplasty, opioid usage was reduced postoperatively. Surgical site injections for femoral fracture and upper extremity procedures showed improved pain and overall increased patient satisfaction [1]. In this article, the role of multimodal analgesia in adult patients undergoing orthopedic surgery is discussed.

## 2. Mechanism of Post-Surgical Pain

**Figure 1:**
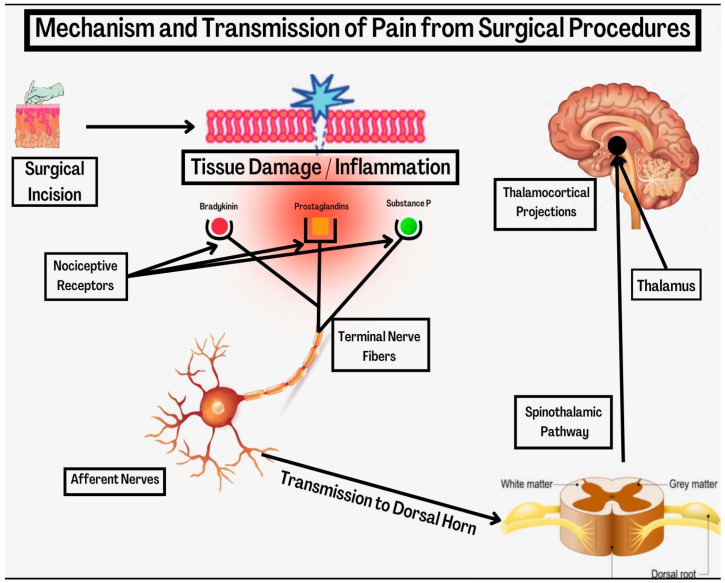
Mechanism and transmission of pain from surgical procedures.

The generation of pain after surgical procedures is complex. Studying pain physiology and transmission is important in understanding its treatment. Depending on how it is transmitted, pain can be classified as nociceptive or neuropathic. Tissue damage results in the activation of nociceptive receptors which are present in the terminal nerve fibers [11]. From there, signals are transmitted through afferent neurons to the dorsal horn, and then through the ascending spinothalamic pathways and thalamocortical projections to higher neural centers in the cerebral cortex [12]. Once there, the pain experience is dictated by the thalamus and cortical modulation. Figure 1 displays the mechanism and transmission of pain signals from tissue injury. This experience is affected by the patient’s personality, sex, emotional state, pain behavior and their culture. Further modulation occurs in the medulla, descending pathways, and dorsal horn of the spinal cord.

Neuropathic pain is the result of direct injury to the nervous system whether it is a peripheral nerve, a nerve root, or spinal cord. It is mediated mostly by A delta and C fibers, but also larger A beta fibers [9]. These fibers innervate both skin and internal organs, including the periosteum, joints, viscera, and muscles. The large A beta fibers often cause responses that are out of proportion to the intensity of stimulus such as allodynia, hyperpathia and dysesthesia [9].

A wide variety of chemicals can directly stimulate or sensitize peripheral nociceptors. These chemicals, or ligands, are each associated with a neural receptor, and each can act as a neurotransmitter. They are released in response to tissue injury and increase the intensity of neuronal stimulation or sensitization or cause local extravasation of inflammatory mediators [12]. These agents include bradykinin, prostaglandin, serotonin, histamine, acetylcholine, potassium ions, and hydrogen ions.

Bradykinin, potassium, and acetylcholine directly activate nociceptors. Prostaglandins do not directly activate nociceptors but are very effective in sensitizing them to further activation by other chemicals [12]. Neurogenic inflammation results because action potentials in an activated neuron travel not only from the periphery to the central nervous system but also antidromically to the periphery, resulting in the release of neuropeptides from the nociceptive terminal [13]. Substance P is one such vasoactive neuropeptide that causes local vasodilation and extravasation. It is released by local free nerve endings around the site of injury and is responsible for further release of bradykinin [14]. Substance P also stimulates the release of histamine and serotonin. Prostaglandins are produced by the action of phospholipase A. Phospholipase A may be stimulated by endogenous chemicals, such as catecholamines, and by local trauma.

If the peripheral pain stimulus is intense and of sufficient duration, the phenomenon of secondary hyperalgesia (hypersensitivity of the area surrounding the initial injury site) and central sensitization may occur. Hyperalgesia is thought to be mediated through the N-methyl-D-aspartate (NMDA) receptor [9]. Central sensitization or wind up is a phenomenon where low threshold benign sensory input evokes a response from nociceptive receptors which normally respond only to painful stimulus. Once it is established, this is difficult to treat.

## 3. Role of the Anesthesiologist

The role of the anesthesiologist as a perioperative physician in the management of orthopedic patients cannot be overemphasized. Even as patients are preparing for surgery, optimizing patients with input from anesthesiologists is important. In addition to addressing general health conditions, weaning patients preoperatively from opioids seems to improve the outcomes in patients, especially those undergoing total joint replacements. The anesthesiologist plays a central role, ensuring accelerated and safe recovery of the patient from surgery. Using techniques which minimize or eliminate the need for general anesthesia decreases the risk of nausea, vomiting and shortens recovery period. Using regional anesthetic techniques successfully where applicable, as a part of multimodal analgesia is a testimony to the skill of the anesthesiologist. Adequate pain control aids in recovery and increases overall patient satisfaction while decreasing the overall cost of care.

## 4. Methods of Analgesia

Multimodal analgesia is defined as the use of more than one modality of pain control to achieve effective analgesia while reducing opioid-related side effects [15]. Traditionally, the treatment of postoperative pain mainly consisted of the administration of opioids which was not only unsatisfactory, but often resulted in multiple side effects including, respiratory depression, ileus, nausea and vomiting, sedation, and urinary retention which impeded recovery. Prolonged dependency on these medications led to chronic addiction. As the understanding of pain mechanisms has improved, the development of different groups of medications to address pain has also advanced. Multimodal analgesia is based on the principle that combining analgesics with different modes or sites of action will result in better pain control and less side effects through opioid sparing. When used as a part of enhanced recovery protocols, this implementation will accelerate functional recovery, decrease hospital costs, and improve patient satisfaction and outcomes.

### 4.1. Pre-Emptive Analgesia

Pre-emptive analgesia refers to administration of pain medications or performing procedures before the surgical incision to prevent surgical pain. It is more effective than the same intervention when started after surgery [16]. Pre-emptive analgesia is intended to prevent peripheral and central hypersensitivity, decrease the incidence of hyperalgesia, and reduce the intensity of postoperative pain [17]. The concept was originally introduced by Crile, over a century ago, based on clinical observation and was later studied by Wall and Woolf [18,19]. The concept was based mainly on animal studies and later human studies followed. Pre-emptive analgesia has a protective effect on the nociceptive system, which could significantly reduce the level of pain and decrease the risk for the development of chronic pain [19]. A combination of Cox-2 inhibitors and pregabalin, given between half an hour and one hour before the surgery, seems to decrease the intensity of postoperative pain after total joint replacements and enable faster recovery. Administration of Ketamine prior to incision, sometimes followed by an infusion, also significantly reduced the need for pos- operative pain medications and seems to play a role in decreasing development of chronic pain by preventing neuromodulation. Performing peripheral nerve blocks where possible prior to the surgical incision decreases the need for supplemental pain medications during and after the surgical procedure. Although initial clinical trials have been inconclusive, the concept of pre-emptive analgesia is an important part of multimodal analgesia regimens.

### 4.2. Surgical Site Infiltration

Infiltration of local anesthetics sometimes in combination with other medications has been one approach used by surgeons to prolong the duration of local analgesia. These medications work by directly preventing the generation and conduction of pain signals from the incision site [20]. Intra-articular injections of slow-release formulations of bupivacaine have been shown to extend the duration of pain relief and decrease the need for supplementation [20]. Newer combinations of local anesthetics with anti-inflammatory properties are also being developed. Each of these techniques is operator dependent and there might be a variation in the success rate. The risk of toxicity when administering large doses of local anesthetics and a potential for chondrolysis when injected into the joints have limited the use of these techniques [20]. Nonetheless, surgical site infiltration can be a good adjuvant to systemic analgesics and forms an important part of multimodal analgesia.

### 4.3. Analgesic Techniques

Analgesic techniques can be pharmacological or non-pharmacological. Pharmacological techniques include central neuraxial techniques, regional anesthesia, and systemically administered medications. The non-pharmacological techniques involve acupuncture, aromatherapy, music therapy, application of hot and cold compresses, elevation of surgical extremity, transcutaneous nerve stimulation, peripheral nerve stimulation and hypnosis.

### 4.4. Central Neuraxial, Regional and Local Analgesia

The use of epidural analgesia has been shown to reduce the requirement for opioids. It has been proven to decrease the surgical stress response, thromboembolic phenomenon, cardiac arrhythmias, pulmonary complications (atelectasis, pneumonia) and earlier return of bowel function [21]. Spinal analgesia with low doses of local anesthetics has also been shown to be effective. Both techniques can be used as a part of multimodal analgesia. Epidural administration is not suitable for outpatient procedures and when early ambulation is desired [21]. Caution should be exercised when central neuraxial techniques are used in combination with antithrombotic agents.

### 4.5. Regional Anesthesia

Use of regional nerve blocks as adjuvants for pain management has evolved into the most favored approach, especially in orthopedic surgeries involving upper extremities [22]. Brachial plexus nerve blocks via interscalene, axillary, supraclavicular and infraclavicular approach, when performed under ultrasound guidance are highly successful. Other nerve blocks at the elbow, wrist, and digital nerve blocks provide ideal pain relief for certain procedures.

For surgeries involving the lower extremity, lumbar plexus block, femoral nerve block, adductor canal block, and popliteal blocks are also very effective for providing regional analgesia [22]. Newer blocks such as Infiltration Between Popliteal Artery and Capsule of the Knee (iPACK), Pericapsular Nerve Group Block 4 (PENG), and Erector Spinae Plane (ESP) performed by experienced anesthesiologists under ultrasound guidance are also very effective in controlling post-operative pain.

### 4.6. Patient Controlled Analgesia

Patients can control the dose of analgesia they receive through a programmable pump. The pump limits the maximum dose, and hence, decreases the chance of dangerous side effects [23]. A continuous infusion, often combined with boluses, can be used for control of postoperative pain. The most commonly used medications are morphine, fentanyl, and hydromorphone, along with local anesthetic agents. Patients are prone to have the same side effects as parenteral opioids, such as nausea, vomiting, pruritus, drowsiness, ileus, and urinary retention; therefore, these pumps have fallen out of favor [23].

## 5. Systemic Analgesics

Systemic analgesics can be administered intravenously (IV), intramuscularly (IM), or orally (PO). In the immediate post-operative period, IV medications are administered to get pain under control. Oral medications are administered as early as feasible, keeping in mind the time taken for onset of action. As a part of enhanced recovery protocols, some oral pain medications are administered pre-emptively before the surgery, for analgesia to take effect by the time anesthesia wears off. The goal is to minimize the administration of IV pain medications to decrease the risk of adverse effects [24]. These medications include opioids and non-opioids such as acetaminophen, non-steroidal anti-inflammatory drugs (NSAIDs), N-methyl-D-aspartate (NMDA) receptor antagonists, anticonvulsants (e.g., gamma-aminobutyric acid (GABA) analogues), beta-blockers, alpha-2 agonists, transient receptor potential vanilloid receptor agonists (capsaicin), glucocorticoids and magnesium. Please refer to the tables below for more information regarding these classes of medications.

### 5.1. Opioids

Opioids have been the mainstay for the management of postoperative pain for many years. Their use is limited by side effects such as nausea and vomiting, respiratory depression, ileus, pruritus, and urinary retention, along with carrying a dangerous risk of addiction. With efficient use of nonopioids, opioids can be mainly used as rescue medications when pain is not well controlled with other methods. Table 1 highlights the most commonly used medications for orthopedic surgeries.

### 5.2. Acetaminophen

Acetaminophen is an effective analgesic for mild to moderate pain. When used as an opioid adjunct, oral or rectal acetaminophen reduces pain intensity and opioid consumption by up to 30% [27,28]. Both acetaminophen and NSAIDs act by inhibiting prostaglandin synthesis. Acetaminophen has a very favorable safety profile and adverse effects are rare [29]. Table 2 highlights this medication for orthopedic surgeries.

### 5.3. Non-Steroidal Anti-Inflammatory Drugs (NSAIDs)

NSAIDs can be classified as specific cyclooxygenase (COX) 2 inhibitors and nonspecific inhibitors of COX 1 and COX 2. COX 1 inhibitors are associated with increased risk of surgical site bleeding, gastrointestinal ulceration, and renal dysfunction [30]. COX 2 inhibitors have fewer side effects and have been shown to decrease opioid requirements and have been shown to contribute to a quicker recovery after surgery [31]. Table 2 highlights this class of medications for orthopedic surgeries.

### 5.4. N-Methyl D-Aspartate (NMDA) Receptor Antagonists

NMDA receptors are involved in the development of pathological pain states such as hyperalgesia and development of chronic pain. Ketamine is a NMDA antagonist which when given before the incision has been shown to decrease the opioid requirement and development of chronic postsurgical pain [32]. Other NMDA receptor antagonists include dextromethorphan, memantine, and magnesium sulfate which have less predictable actions. NMDA receptor antagonists have potentially undesirable side effects, such as psychosis, especially in susceptible patients who have a history of psychiatric disorders. Nonetheless, in low doses, they have been used as a part of multimodal analgesia without side effects as aids to pain management [32]. Table 2 highlights this medication for orthopedic surgeries.

**Table 2:** Acetaminophen, nonselective and selective NSAIDs, COX-2 inhibitors, and NMDA receptor antagonists [25,33].

Drug Class	Dose	Analgesic Duration	Metabolism	Comments
Para-aminophenol Derivative				
Acetaminophen	650–1000 mg IV	4–6 h	- Liver—Glucuronidation, Sulfation, and CYP450 oxidation - Active metabolites are renally excreted	- Lacks anti-inflammatory activity - Avoid in severe hepatic diseases
**Nonselective NSAIDs**				
Ketorolac	15–30 mg IV	4–6 h	- Liver—Hydroxylation and Conjugation - Products of metabolism are excreted renally	- Maximum dose 120 mg/day - Reduced opioid consumption 25–45% - Contraindicated in coronary artery bypass graft surgeries
Ibuprofen	400–800 mg IV	4–6 h	- Liver—CYP450 - Products of metabolism are excreted renally	- Maximum dose 3200 mg/day - Contraindicated in coronary artery bypass graft surgeries
**Selective COX-2 Inhibitor**				
Parecoxib	20–40 mg IV	6–12 h (IV)	- Liver—CYP3A4 and CYP2C9 to valdecoxib	- Dose reduction needed for older adults and weight < 50 kg - Contraindicated in coronary artery bypass graft surgeries
**N-methyl D-asparate (NMDA) Receptor Antagonist**				
Ketamine	0.5–1 mg/kg/h IV	30–60 min IV	- Liver—CYP3A4 and CYP2C9 to valdecoxib	- Acts on mu/delta receptors - Phencyclidine (PCP) derivative, associated with delirium and hallucinations - Contraindicated in history of cerebrovascular accidents, can increase intracranial pressure

### 5.5. Anticonvulsants (Gamma-Aminobutyric Acid Analogues)

Gabapentin and pregabalin are GABA analogues that can be administered orally. They can be used before and after surgical procedures and have been shown to decrease the requirement for narcotic pain medications. The disadvantages of GABA analogues are their adverse effects, including sedation, visual disturbances, dizziness, and headache [34]. Table 3 highlights this class of medications for orthopedic surgeries.

### 5.6. Beta-Blockers

Use of beta-blockers, such as short-acting esmolol, intraoperatively not only blunts the cardiovascular response to surgical stimulus, such as tachycardia and hypertension, resulting in the decreased incidence of adverse cardiac events, but also decreases analgesic requirements postoperatively due to its anti-nociceptive effect [36]. Table 4 highlights this class of medications for orthopedic surgeries.

### 5.7. Alpha-2 Agonists

Clonidine and Dexmedetomidine are systemic alpha-2 agonists that can decrease postoperative opioid consumption, pain intensity, and opioid-related side effects (i.e., nausea). These medications are often added to an opioid-based regimen [37]. These medications can be administered at various times and via several different routes of administration. Clonidine can be administered orally, intravenously, and transdermally as well. Dexmedetomidine is usually given as an intravenous infusion started prior to wound closure. Table 4 highlights this class of medications for orthopedic surgeries.

**Table 4:** Beta adrenergic receptor blockers, alpha 2 adrenergic receptor agonists [25,38].

Drug Class	Dose	Duration of Hemodynamic Effect	Metabolism	Comments
Beta Adrenergic Receptor Blockers				
Atenolol	25–50 mg PO	12–24 h (PO)	- Limited liver metabolism	- Caution in patients with bronchospastic disease - Can be used in pediatric populations with prolonged QT syndrome - Avoid in patients with myasthenia gravis
Metoprolol	100 mg PO 2.5–5 mg IV	3–6 h (PO)3–4 h (IV)	- Liver—CYP2D6	- Caution in patients with bronchospastic disease - Avoid in patients with myasthenia gravis - Found in breast milk and crosses placenta
Esmolol	0.5–1 mg/kg (IV)	10–30 min (IV)	- Blood hydrolysis of methyl ester linkage	- Can decrease tachycardia and hypertensive emergency - Contraindicated in severe sinus bradycardia - Utilized primarily intraoperatively
**Alpha-2 Adrenergic Receptor Agonists**				
Clonidine	0.1 mg PO	4–6 h	- Liver to inactive metabolites	- Epidural clonidine may produce pain relief at spinal presynaptic and postjunctional alpha-2 adrenoreceptors - Avoid in patients with severe coronary insufficiency, chronic renal impairment, and recent myocardial infarction
Dexmedeto-midine	1 mcg/kg IV over 10 min with 0.2–0.7 mcg/kg/hr continuous IV	2–3 h (IV)	- Liver—Glucuronidation, methylation, and CYP2A6	- Monitor for bradycardia and hypotension - Elderly use can exacerbate cardiovascular events - Can see transient paradoxical hypertension due to initial vascoconstrictive effects

### 5.8. Capsaicin (Transient Receptor Potential Vanilloid Receptor 1 (TRPV1) Agonist)

Capsaicin selectively stimulates unmyelinated C-fiber afferent neurons, causing the continued release and subsequent depletion of substance P, which ultimately decreases C-fiber activation [39]. It must be instilled at the surgical site at the time of application [40]. Table 5 highlights this class of medications for orthopedic surgeries.

### 5.9. Lidocaine Infusion

Lidocaine was initially used in the 1940s to treat neuropathic pain in patients with burns. It acts by modifying the expression of sodium channels. An intravenous infusion of lidocaine utilizes its anesthetic properties by reducing circulatory inflammatory cytokines, reducing secondary hyperalgesia and central sensitization [42]. Due to its mechanism of action, lidocaine has an increased risk of cardiac and neurological side effects. Table 6 highlights this class of medications for orthopedic surgeries.

### 5.10. Glucocorticoids

Glucocorticoids decrease post-operative pain and post-operative nausea and vomiting. They exert their analgesic effect via several mechanisms; they have anti-nociceptive effects at the spinal level, prevent the production of cytokines involved in inflammatory pain, and inhibit the production of inflammatory prostaglandins and leukotrienes by preventing arachidonic acid production [16,44]. In high doses, they have been known to cause hyperglycemia, which is not significant at the usual doses used. For orthopedic surgeries involving joints, spinal facet joints are injected with a 1:1 ratio of corticosteroid to anesthetic. Extremity joints, such as the elbow or wrist, require 2–4 ccs of solution, while larger joints, such as the hip, knee, or sacroiliac, require 4–8 ccs of solution, containing the corticosteroid and anesthetic [44]. Table 7 highlights this class of medications for orthopedic surgeries.

### 5.11. Magnesium

Intravenous magnesium has been shown to have an opioid-sparing effect, with a reduction in pain scores. It is administered as a bolus dose of 30–50 mg/kg IV, followed by 6–20 mg/kg/hr infusion for 4 h. Extracellular magnesium blocks the NMDA receptor in a voltage-dependent manner [45], and thus, can prevent the establishment of central sensitization. Table 7 highlights this class of medications for orthopedic surgeries.

## 6. Conclusions

Pain is a subjective sensation and is difficult to assess. A variety of pain assessment tools such as the VAS (verbal analog score), the McGill Pain Questionnaire, and a brief pain inventory can be used. The Wong–Baker facial grimace scale is useful when patients are unable to communicate their pain intensity. The majority of postoperative pain is nociceptive with a small neuropathic component. Pain management strategies should address both types of pain. In the words of Dr. John Bonica, the father of pain medicine, “Pain is what a patient says it is.” Effective pain control requires constant reassessment with individualized strategies.

## Figures and Tables

**Table 1:** Opioids [25,26].

Drug Class	Dose	Analgesic Duration	Metabolism	Comments
Parenteral Opioids				
Morphine	2–4 mg IV	3–4 h	- Liver—glucuronidation - Active metabolites are renally cleared	- Causes Histamine release, can induce vagally mediated venodilation, hypotension, bradycardia
Hydromorphone	0.5–1 mg IV/SC	2–4 h	- Liver—glucuronidation	- Can be used in renally impaired patients - Avoid in patients suffering from hypovolemic shock - Contraindicated in Genitourinary Obstructions
Fentanyl	1–2 mcg/kg IV	30 min–1 h	- Liver—CYP3A4 and CYP3A5	- Up to 100× more potent than morphine - Can be used in renally impaired patients - Can be used in patients with hemodynamic instability or bronchospasm
Meperidine	50–150 mg IV	2–3 h	- Liver—CYP3A4 and CYP3A5	- Active metabolite, normeperidine, can cause seizures - Contraindicated with monoamine oxidase inhibitors - Elderly have a slower elimination rate
**Oral immediate-release opioids**				
Codeine	15–60 mg PO	4–6 h	Liver—CYP2D6	- Avoid in pediatric patients - Causes increased intracranial pressure
Hydrocodone	2.5–10 mg PO	4–6 h	Liver—CYP2D6 and CYP3A4	- Avoid in pediatric patients - Causes increased intracranial pressure
Oxycodone	5–10 mg PO	3–6 h	Liver—CYP2D6 and CYP3A4	- Avoid with CYP3A4 inhibitors (macrolide antibiotics/azole-antifungal agents) - Causes increased intracranial pressure
Tramadol	50–100 mg PO	3–6 h	Liver—CYP2D6 and CYP3A4	- Avoid with CYP3A4 inhibitors (macrolide antibiotics/azole-antifungal agents)
Oxymorphone	5–10 mg PO	3–6 h	None known	

**Table 3:** Anticonvulsants [25,35].

Drug Class	Dose	Analgesic Duration	Metabolism	Comments
Anticonvulsants				
Gabapentin	300 mg PO	5–7 h	- Kidney—First-order kinetic elimination	- Avoid in patients with renal impairment - Contraindicated in patients with suicidal ideations - Avoid in patients with seizure disorders and substance abuse
Pregabalin	100 mg PO Q8Hr	12 h		- Has a 6× higher binding affinity for GABA than Gabapentin - Caution in patients with renal impairment - Avoid in patients with seizure disorders and substance abuse

**Table 5:** TRPV1 receptor agonist [41].

Drug Class	Dose	Analgesic Duration	Metabolism	Comments
Transient Receptor Potential Vanilloid Receptor (TRPV1) Agonist				
Capsaicin	0.025% patch	- Could potentially last several weeks	Liver—CYP2E1	- Do not use on wounds, damaged, broken, irritated skin, - May cause serious burns (1st- to 3rd-degree chemical burns), especially during application of patch - Cannot use in combination with external heat sources

**Table 6:** Local anesthetics [42,43].

Drug Class	Dose	Analgesia Duration	Metabolism	Comments
Local Anesthetics				
Lidocaine	5 mg/kg plain 7 mg/kg with epinephrine	30–90 min	- Liver—CYP1A2, CY2A6, and CYP3A4 - Active metabolites can accumulate and may cause CNS toxicity	- Peripheral Nerve Block: 2% Lidocaine acts in 10–20 min, with 3–8 h of analgesia - Spinal: 1–2% Lidocaine acts in 15 min, with ~2–3 h of anesthesia - Contraindicated in patients with Wolff-Parkinson-White syndrome - Use in extreme caution in patients with pseudocholinesterase deficiency
Mepivacaine	7 mg/kg plain 8 mg/kg with epinephrine	1–2 h	- Liver—demethylation, hydroxylation, and glucuronidation - Renally excreted	- Peripheral nerve block: 1.5% mepivacaine acts in 10–20 min, with 3–10 h of analgesia - Has been associated with methemoglobinemia - Avoid in patients with G6PDH deficiency
Chloroprocaine	14 mg/kg max 1000 mg with 1:100,000 epinephrine 11 mg/kg max 800 mg plain for peripheral nerve blocks	40–60 min	- Rapidly hydrolyzed by plasma pseudocholinesterase	- Has been associated with methemoglobinemia - Avoid in patients with G6PDH deficiency
	50 mg for intrathecal	Spinal 1–1.5 h		
Bupivacaine	2.5 mg/kg plain		- Liver—CYP3A4, CYP2D6, CYP2C19	- Use with caution in patients with heart block - Has direct vasodilating properties, which can exacerbate cardiovascular collapse
	3 mg/kg with epinephrine	6–8 h		
	Spinal-1–2 mL of 0.75% bupivacaine	2–4 h		
Ropivacaine	3 mg/kg	1.5–8 h	- Liver—CYP1A2, CYP3A4, and CYP2D6 - Inactive metabolites are renally excreted	- Peripheral Nerve Block: 0.5% Ropivacaine has 15–30 min onset, with duration of analgesia 5–24 h

**Table 7:** Glucocorticoids and Magnesium [44,45,46].

Drug Class	Dose	Analgesia Duration	Metabolism	Comments
Glucocorticoids				
Methylprednisolone	40–120 mg	~2 weeks	- Liver—CYP3A4 and renal excretion	- Contraindicated in patients with idiopathic thrombocytopenic purpura - Cannot be given intrathecally
Triamcinolone	5–40 mg	~2 weeks		- Dose is adjusted based on size of joints (smaller size requires smaller dose) - Contraindicated in patients with idiopathic thrombocytopenic purpura
Dexamethasone	8–24 mg (IV)	~48 h		- Contraindicated in patients with severe hypertension, gastritis, or chronic heart failure - Has a large substantial role in reducing post-operative nausea and vomiting
**Magnesium**				
Magnesium	30–50 mg/kg (IV)	Enhances analgesic effect of other medications	- Absorbed in the jejunum and ileum	- Extracellular magnesium blocks NMDA receptor in a voltage-dependent manner - Can cause decreased deep tendon reflexes

## Data Availability

Not applicable.
